# Ultramodern natural and synthetic polymer hydrogel scaffolds for articular cartilage repair and regeneration

**DOI:** 10.1186/s12938-025-01342-3

**Published:** 2025-02-07

**Authors:** Chun-Sheng Li, Yan Xu, Juan Li, Shu-Hao Qin, Shao-Wen Huang, Xue-Mei Chen, Yi Luo, Cheng-Tao Gao, Jian-Hui Xiao

**Affiliations:** 1 Institute of Medicinal Biotechnology, Affiliated Hospital of Zunyi Medical University, 149 Dalian Road, Huichuan District, Zunyi, 563003 China; 2 National Engineering Research Center for Compounding and Modification of Polymer Materials, Guizhou Material Industrial Technology Research Institute, Guiyang, 550014 China; 3 Guizhou Provincial Key Laboratory of Medicinal Biotechnology and Research Center for Translational Medicine in Colleges and Universities, Affiliated Hospital of Zunyi Medical University, 149 Dalian Road, Huichuan District, Zunyi, 563003 China; 4 Department of Pediatrics, Affiliated Hospital of Zunyi Medical University, 149 Dalian Road, Huichuan District, Zunyi, 563003 China

**Keywords:** Articular cartilage injury, Cartilage tissue engineering, Hydrogel scaffold, Repair and regeneration

## Abstract

Articular cartilage injury is a serious bone disease that can result in disabilities. With the rapid increase in the aging population, this disorder has become an increasingly important public health issue. Recently, stem cell-based cartilage tissue engineering has emerged as a promising therapeutic option for treating articular cartilage damage. Cellular scaffolds, which are among three key elements of tissue engineering, play significant roles in the repair of damaged articular cartilage by regulating cellular responses and promoting cartilage tissue regeneration. Biological macromolecules are commonly used as scaffold materials owing to their unique properties. For example, natural and synthetic polymer hydrogel scaffolds can effectively mimic the microenvironment of the natural extracellular matrix; exhibit high cytocompatibility, biocompatibility, and biodegradability; and have attracted increasing attention in bone and cartilage tissue engineering and regeneration medicine. Several types of hydrogel scaffolds have been fabricated to treat articular cartilage abnormalities. This article outlines the recent progress in the field of hydrogel scaffolds manufactured from various biomaterials for repairing damaged articular cartilage, discusses their advantages and disadvantages, and proposes directions for their future development.

## Introduction

Articular cartilage is a highly specialized tissue devoid of blood, nerves, and lymph and primarily composed of collagen, proteoglycans, and chondrocytes, which cannot easily self-repair in the event of an injury [1]. Articular cartilage injuries (ACI) are caused by the lack of classical healing cascade responses in articular cartilage, such as coagulation, blood infiltration, inflammation, and senescent cell accumulation and external factors, such as aging, obesity, and trauma. ACI result in the biochemical breakdown of articular cartilage, which can lead to traumatic arthritis, osteoarthritis (OA), and ultimately progressive total joint destruction [2–4]. The limited regenerative capacity and poor self-healing ability of articular cartilage are the primary reasons for its susceptibility to degenerative lesions and defects [5]. Common cartilage injury types include cartilage wear and articular cartilage tears and denudation. The morbidity rate of ACI is ~ 36%, which is affected by concomitant ligament, meniscus, or soft tissue injuries; however, very few studies were devoted to ACI treatment options [6], and an effective ACI treatment procedure has not been developed yet. Recently, stem cell-based bone and cartilage tissue engineering has emerged as a promising therapeutic approach for ACI treatment. Cellular scaffolds composed of different biomaterials play a significant role in bone and cartilage tissue engineering. Among these bio-scaffold materials, hydrogels have good application prospects because of their biocompatibility, safety, and ability to promote chondrogenesis.

Hydrogel is a three-dimensional (3D) network comprising chemically or physically bonded crosslinks of hydrophilic polymers [7]. They represent a viable alternative to the natural extracellular matrix (ECM) because of their good biocompatibility, tissue adhesion, biodegradability, sustained release of drug-carrying agents, high water content, flexibility, and biomimetic design [8–10]. Hydrogels primarily include natural and manufactured polymeric components. Hyaluronic acid (HA), collagen, chitosan, silk fibroin, alginate, agarose, gelatin, and other naturally occurring biological materials exhibit good biocompatibility and degradability [11]. Polyethylene glycol (PEG), polylactic acid (PLA)–co-glycolic acid (PLGA), polycaprolactone (PCL), poly(d-lactic acid), poly(dl-lactic acid) (PDLLA), polycarbonate, poly(*N*-isopropyl acrylamide), and polyvinyl alcohol (PVA) are synthetic biomaterials with relatively high stability [12] whose biological properties can be enhanced by combining them with natural or synthetic biomaterials. For example, composite chitosan hydrogels reinforced with hydroxyapatite nanorods can promote secondary crosslinking to endow the hydrogel network with high deformation rate and compressive strength, excellent self-recovery properties, and high fatigue resistance [13]. Without changing the crystallinity of silk gelatin or PVA, silk gelatin/PVA composite hydrogels can significantly increase the thermal stability of silk gelatin and improve its mechanical properties [14]. Recently, hydrogels have been successfully used to repair cartilage injuries and demonstrated good ACI therapeutic properties [15]. In particular, the use of hydrogel-encapsulated mesenchymal stem cells (MSCs) for treating articular cartilage damage has found to be highly efficient, because it enables targeted distribution, increases retention at the injury site, and improves the MSC functionality [16]. Furthermore, hydrogels that are produced from different materials exhibit various biological properties and therapeutic potentials. To optimize ACI treatment approaches, hydrogels for cartilage repair fabricated from various materials should be thoroughly reviewed.

**Fig. 1:**
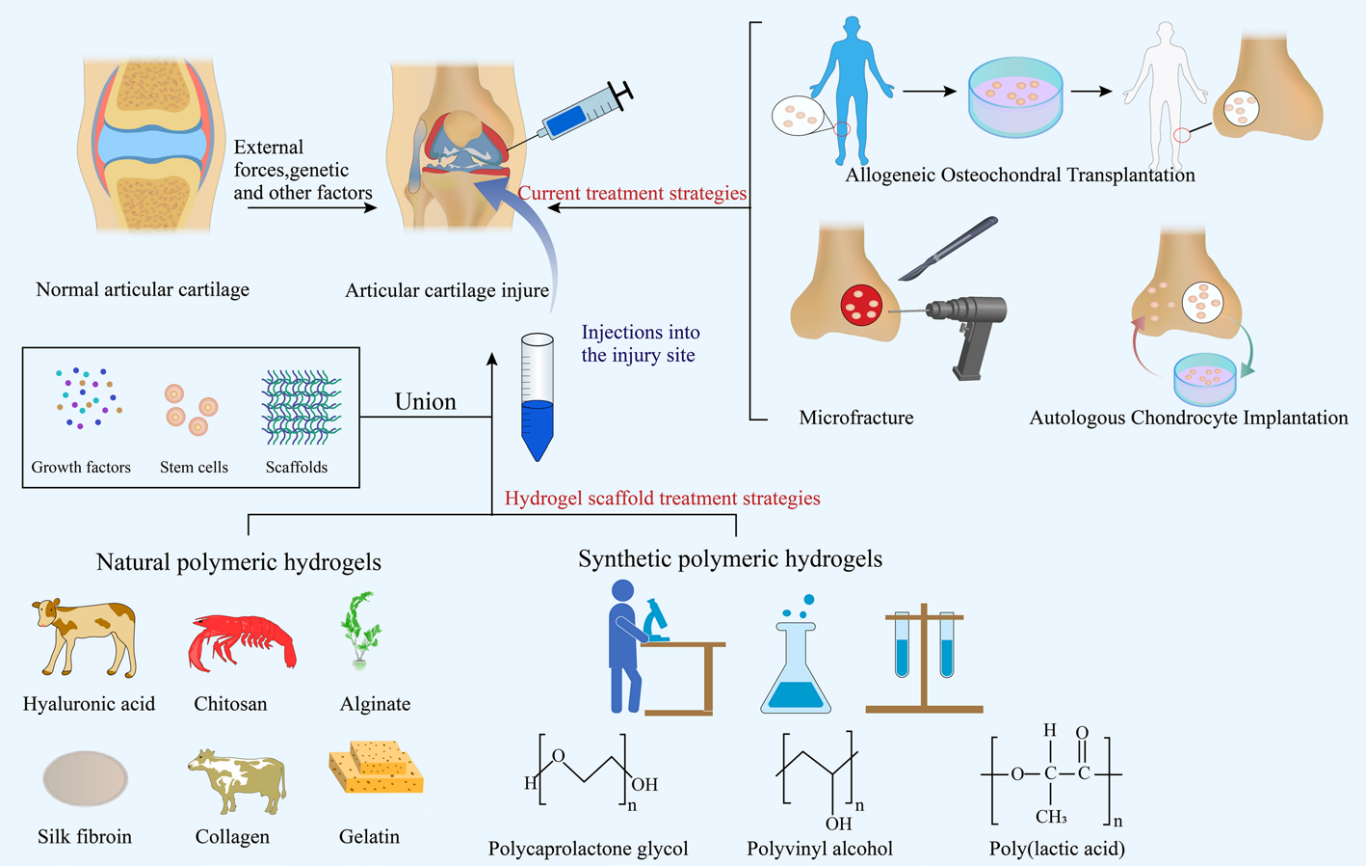
Treatment strategies for articular cartilage injuries. Current clinical treatments include microfractures, osteochondral allograft transplantation, and autologous chondrocyte transplantation. The hydrogel-based repair strategy for cartilage injuries involves natural and synthetic polymer hydrogels

Therefore, in this review, we summarize the traditional ACI treatment strategies and focus on the recent progress in the field of hydrogel scaffolds fabricated from various materials for repairing damaged articular cartilage (Fig. 1). Hydrogel materials are classified according to their sources, and the advantages and disadvantages of different hydrogel scaffold materials commonly used in the field of cartilage tissue engineering are discussed.

## Traditional ACI treatments

Traditional ACI treatments include surgical and nonsurgical procedures. Nonsurgical treatments are primarily used to reduce pain in patients via conservative treatments, such as oral and intra-articular injection drugs and physical therapy. Surgical treatments, which are primarily based on reconstructive surgery, are applied to full-thickness cartilage and osteochondral lesions to improve the joint function and consistency and prevent the lesion site from damaging the normal cartilage area [17, 18]. Currently, surgical treatments include microfracture, osteochondral allograft, autologous chondrocyte implantation, and osteochondral auto-transplantation [18]. During these surgical procedures, microfracture, a minimally invasive surgery involving the micro-breakdown of the subchondral bone, can alleviate pain in the short term; however, its long-term effects are limited [19]. Osteochondral transplantation is an invasive surgery that requires not only a large incision but also donor grafts that are not easily available [20]. Osteochondral allografts can damage the donor cartilage during transplantation in a situation, when the graft cannot stably fit the joint surface of the patient. This may result in the generation of excessive stress on the graft edge, infection, or graft loosening, leading to excessive postoperative bleeding and pain at the donor site [21]. Autologous chondrocyte implantation has been gradually improved from the early periosteal-based and collagen-covered to matrix-induced autologous chondrocyte implantation; however, postoperative rehabilitation cannot fully adapt to these new surgical techniques [22]. Certain rehabilitation methods, such as cold therapy and hydrotherapy, remain at the stage of theory or case reports and lack high-quality randomized controlled trials supporting their efficiency. These treatments may temporarily relieve the symptoms of patients; however, fibrocartilage rather than articular hyaline cartilage is formed during postoperative healing. Fibrocartilage is inferior to natural articular cartilage in terms of wear resistance and stress–strength characteristics [23, 24]. In addition, the formation of fibrocartilage can destroy the integrity of the cartilage ECM, promote pathological changes in OA, and cause mineral deposition in articular cartilage or the development of osteophytes. Therefore, traditional ACI treatment approaches, including non-surgical and surgical procedures, have serious limitations.

## Stem cell-based hydrogel scaffold treatment of ACI

With the recent and rapid development of biomedical technology, regenerative medicine and tissue engineering focused on stem cells, growth factors, and scaffolding materials have demonstrated a significant potential for addressing the problems related to inadequate donor sources and immune rejection. Stem cells and growth factors can differentiate cartilage types; however, the direct intra-articular transplantation of MSCs may result in cell dispersion and an insufficient number of inoculated cells for regeneration. In addition, directly implanted MSCs may rapidly degrade owing to harsh mechanical loading and the presence of catabolic factors in the diseased joints [25]. Thus, cell viability and differentiation can be enhanced by physically protecting transplanted cells and providing a suitable microenvironment for regulating signaling molecules via functional carriers. Moreover, they must be retained at the implantation site, integrated with adjacent tissues, and exhibit sufficient porosity to enable the inward growth of host tissues [26, 27].

In 1960, Wicherle and Lim synthesized crosslinked poly (methyl hydroxyethyl acrylate) hydrogels for corneal contact lenses [10], thus pioneering research in the potential biomedical applications of synthetic hydrogels. Stem cell-based cartilage tissue engineering using different hydrogel scaffolds has recently made significant progress in the field of cartilage tissue repair. Hou et al. [28] prepared an injectable supramolecular hydrogel based on dextrose anhydride, encapsulated rabbit bone marrow mesenchymal stem cells (BMSCs) into the hydrogel, and implanted them subcutaneously into nude mice, confirming the regeneration of cartilage tissues. Hydrogel scaffolds has played a key role in this success; however, although such scaffolds can provide a desirable platform for cellular adhesion, survival, differentiation, and function because of their unique physical and chemical properties, which are similar to those of the natural ECM, the physicochemical properties and biological functions of hydrogel scaffolds are closely associated with their origin, composition, and microstructure.

Hydrogels are usually porous materials, making it difficult to create a strong bond between the cells and the scaffold. Cells need to attach, grow and proliferate on the hydrogel surface to obtain sufficient nutrients and space to continue growing. However, it is difficult for cells to attach to the hydrogel surface due to the tension between the free water in the hydrogel and the extracellular matrix. Therefore, we can enhance the mechanical stability of the hydrogel by altering the mechanical shear or chemical cross-linking, and also by physical cross-linking to form a multilayered network or forming a new interlayer network structure between two layers to achieve and thus increase the possibility of cell–hydrogel binding. Researchers modified hydrogels by doping different hydroxyapatite nanofibers (HANFs) to alter the hydrogel structure and improve its mechanical strength and osteoinductivity to some extent. The results showed that the composite hydrogels had good cytocompatibility and the addition of m-HANFs facilitated cell adhesion and growth [29]. Hao Chunxiang’s group [30] prepared an osteochondral integrated multiphase scaffold, which showed that the layers of the scaffold were tightly integrated with no obvious discontinuity or separation from each other in both macroscopic and Micro-CT observations. Scanning electron microscopy showed that the structure of the bone layer was relatively dense, while the structure of the intermediate layer and the cartilage layer was relatively loose, and the pore structure of each layer was interconnected with each other with three-dimensionality. MTT method showed that the murine L929 fibroblasts grew well on the scaffolds, and the green fluorescent protein-labeled rat BMSCs grew uniformly on the scaffolds, which showed that the scaffold materials were non-cytotoxic and had good biocompatibility. Oju Jeon and Eben Alsberg [31] Develop an Alginate Hydrogel Using Photocrosslinking; Adjusting the Concentration of the Hydrogel Improves Adhesion, Spreading, and Proliferation of Human Mesenchymal Stem Cells (hMSCs).

In addition, the immune response is a key factor when implanting hydrogel scaffolds and cellular grafts, and it may have a direct impact on treatment outcomes. In cell transplantation, allogeneic transplantation may lead to immune rejection, and to minimize this risk, autologous cell transplantation, in which the patient's own cells are used, can be used to avoid immune rejection. Osteochondral defect repair was induced in 24 adult minipigs using porcine autologous bone marrow MSCs transplanted in combination with a polyglycolic acid–hyaluronic acid scaffold (Chondrotissue^®^). More hyaline cartilage and less connective tissue production were observed at 90 days postoperatively compared to untreated defects [32]. Histological staining using rabbit autologous nasal chondrocytes (NCs) in combination with alginate hydrogel has also been shown to result in better and more transparent repair tissue at 3 and 6 months postoperatively [33]. It has also been shown that the use of autologous MSC seed grafts shows the same histologic and biomechanical repair as osteochondral autografts [34].

## Natural and synthetic polymeric hydrogel scaffolds for ACI

**Fig. 2:**
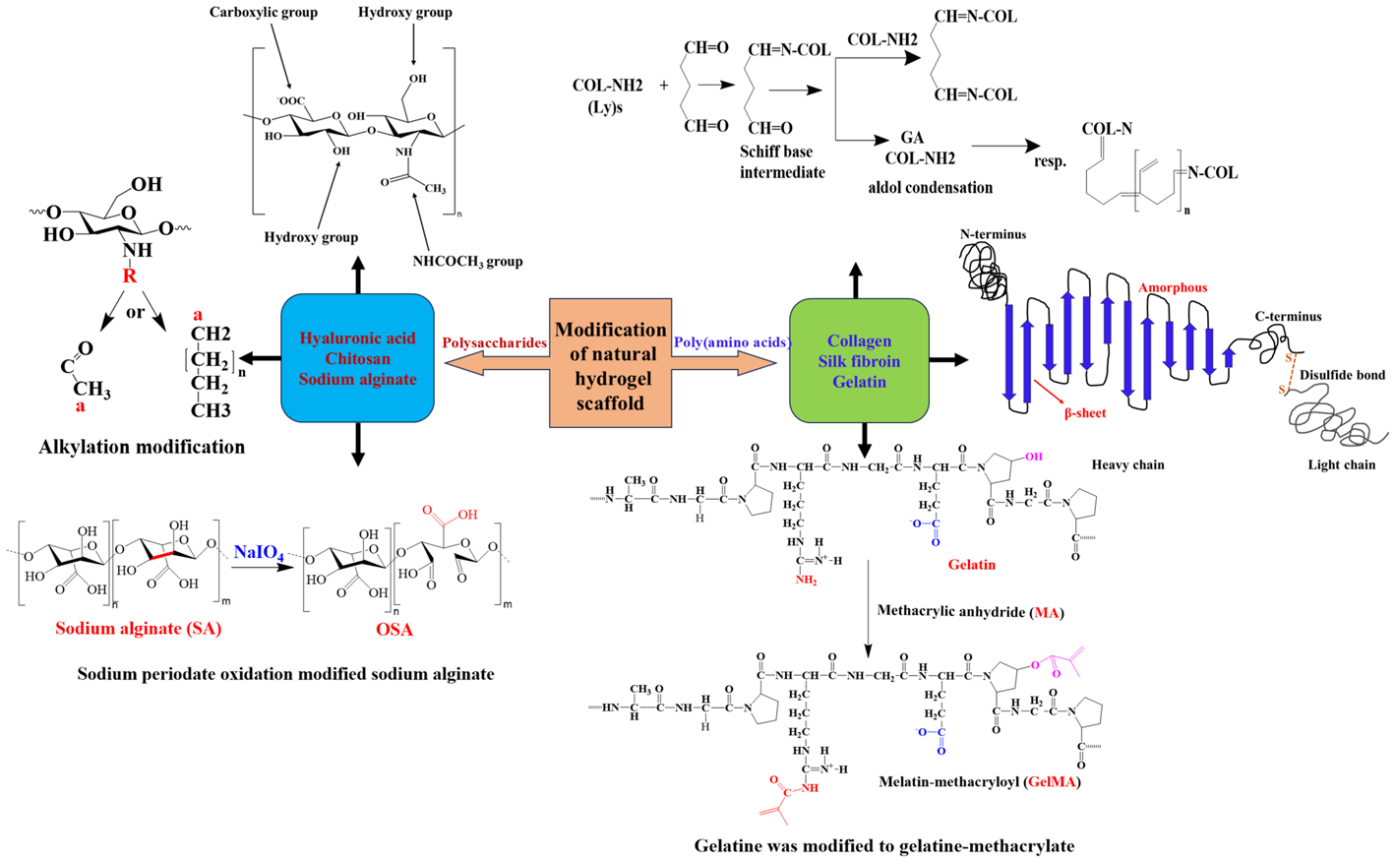
Modifications of different types of natural hydrogel scaffolds

Natural hydrogel scaffold materials are primarily divided into two categories: polysaccharides and poly(amino acids) or proteins. They have excellent biocompatibility and hydrophilicity and low immunogenicity, as outlined in Table 1, thus effectively mimicking the ECM. When present alone or used to transport stem cells, growth factors, or other bioactive compounds, they effectively promote cartilage repair. However, pure natural hydrogel scaffolds have several drawbacks, such as low strength and toughness (Table 1). To overcome these limitations, these scaffolds are often subjected to chemical modifications, crosslinking, or other treatments, as shown in Fig. 2.

**Table 1:** Natural polymeric hydrogel scaffolds

Biomaterials	Chemical structure	Source	Advantages	Disadvantages	References
HA		Produced by the fermentation of *Streptococcus*, extracted from corpus cavernosum, umbilical cord, and synovial vitreous humor, abundant in the ECM of human embryonic and connective tissues	Nourishes, lubricates, and dampens joints; exhibits excellent biocompatibility, cellular affinity, immunomodulatory ability; and inhibits the inflammatory cytokine expression	Poor mechanical properties and high internal degradation rate	[35–38, 43, 44]
Chitosan		Chitin is formed via deacetylation	Biocompatibility, adhesion, and degradability	Low solubility, high viscosity, and low mechanical strength	[46, 47, 51, 52]
Alginate		Obtained from brown algae and *Pseudomonas* mucosa	Highly hydrophilic and water soluble	Poor gel stability, low mechanical strength, rapid drug release, and poor degradation properties	[55–57, 61, 62]
Collagen		Synthesized from fibroblasts and present in organic matter and connective tissues of articular cartilage, tendons, and bones	Physicochemical properties, immunological properties, biodegradability, good biocompatibility, and promotion of cell growth	Insufficient mechanical strength and poor heat resistance	[11, 64–66, 69]
Silk fibroin		Hydrophobic silk cardiac protein and hydrophilic silk glue protein composition	Excellent mechanical properties, biodegradability, biocompatibility, resistance to oxidation, and antibacterial properties	Low mechanical strength	[70, 71, 76]
Gelatin		Present in skin, tendons, ligaments, and bones and extracted via hydrolysis or acid–base reactions	High thermal stability with degradation times up to several weeks. Facilitates ECM deposition and new cartilage tissue formation	Low mechanical strength, poor thermal stability, and high degradation rate	[78, 82, 83]

Synthetic polymeric hydrogel scaffolds differ from natural hydrogel scaffolds in terms of their source materials. They are obtained via chemical synthesis methods, thereby enabling the selection of various polymer monomers and dosages of crosslinking agents to prepare desired hydrogel materials. These synthetic scaffolds exhibit not only the excellent biocompatibility of natural hydrogels but also outstanding mechanical properties and controllable physical and chemical characteristics, as outline in Tables 2 and 3. However, certain synthetic polymers include catalysts, and the chemicals used during their preparation can introduce inherent toxicity to the materials themselves, necessitating rigorous evaluation and management.

**Table 2:** Synthetic polymeric hydrogel scaffolds

Biomaterial	Chemical structure	Source	Advantages	Disadvantages	References
PEG		Polymerized with ethylene oxide	Good biocompatibility and excellent mechanical properties	Chemical composition (–(CH_2_CH_2_O)_n_–), does not contain a biologically identifiable site, and requires modification	[84, 89]
PLA		Lactic acid polycondensation forms aliphatic polymers. Available in two optical forms: poly(d-lactide) and poly(l-lactide)	Good mechanical properties	Low degradation rate, hydrophobic, and low impact toughness	[90, 92, 95]
PVA		Alcoholysis of polyvinyl acetate	Good biodegradability and mechanical properties, non-cytotoxic, and low friction coefficient	Pure PVA hydrogels are also mechanically weak	[97–99, 103]

**Table 3:** Summary of crosslinking modes and properties of natural and synthetic polymer hydrogel scaffolds

Component	Crosslinking method	Strength	Hydration properties	Degradation rate	Aperture/porosity	References
Col/PDA/HA	Freeze drying	800 kPa			200 μm	[40]
HAMA/4-Arm PEG-ACLT	3D bio-printing; UV irradiation					[45]
GCS/DF-PEG	Self-healing	33.19 kPa		18.3%	200–400 μm	[48]
CS-alginate + HAp	TIPS	98–270 MPa			2.93 ± 1.63 × 104 μm^2^	[49]
SH/GO/CS/nHAP	Upper layer SH (EDC and NHS); upper layer CS/GO (ultrasonicated); SH/GO/CS/nHAP(freeze-dried)	Upper layer (0.28 ± 0.024 MPa); upper layer (0.38 ± 0.021 MPa)	High water absorption	15.1%	The upper layer (40–200 µm); the lower layer (30–100 µm)	[50]
CS-KGN/OSA	Room-temperature self-crosslinking	300 kPa	The water content is relatively low	7.8%	20 μm	[53]
HAChCS	LDI crosslinking	0.5–0.8 MPa	High water absorption	40 d (40%)	Macroporosity (≈200 μm) and microporosity (≈10 μm)	[54]
Naringin–BG hydrogels	Mix BG/SA solution and naringin/AG/SA solution					[58]
OSA–DA	Schiff base reduction method and oxidation of NaIO_4_					[62]
US–SF	Ultrasonication	90.95–6.04 kPa	High water absorption	28 d (30%)	Porosity of 86%	[73]
MCS/MSF	UV irradiation	0.32 ± 0.07 MPa	8.79 ± 0.71		The porosity is slightly smaller	[75]
PEG [PRGF]	PRGF (freeze thawing); PEG [PRGF] (Room-temperature incubation)			9–10 d		[86]
PEGDA + GelMA/Alginate	Layered 3D printing PEGDA; PEGDA and GelMA/Alginate low-temperature crosslinking	10–14 MPa	High moisture content		The surface pore morphology is uniform	[93]
PLGA–g-(PCL_5_)_3_	(PLGA + oligo-PCL_5_) TEG was added and mixed evenly, lyophilization	Greater than 0.60 MPa		20% degradation in seven weeks	237 ± 31 μm	[96]
BC–PVA–PAMPS	Freeze thawing; UV irradiation PAMPS	23.0 MPa				[101]
PVA–PMEDAH	PVA and PMEDAH mix; Freeze thawing				Diameters ranging from 1 μm to 300 nm	[102]
PVA/CS	Freeze thawing		The water content is above 85%		200–400 μm	[103]
PVA–PAAc	Freeze thawing; annealing		Fequilibrium water content 48 ± 2%			[104]

*CS* chitosan, *HAp* hydroxyapatite, *GO* graphene oxide, *SH* sodium hyaluronate, *EDC* 1-ethyl-3-(3-dimethylaminopropyl), *NHS* *N*-hydroxysuccinimide, *TIPS* thermally-induced phase separation, *KGN* kartogenin, *OSA* aldehyde-modified oxidized alginate, *ADH* adipic dihydrazide, *HACh* hyaluronic acid–chitosan scaffolds, *LDI* l-lysine diisocyanate, *BG* bioglass, *SA* sodium alginate, *AG* agarose, *DA* dopamine hydrochloride, *SF* silk fibroin, *US* ultrasonic, *MSF* methacrylated silk fibroin, *MCS* maleilated chitosan, *BA* benzyl alcohol, *TEG* triethylene glycol, *BC* bacterial cellulose, *PAMPS* poly(2-acrylamido-2-methyl-1-propanesulfonic acid sodium salt), *pMEDSAH* poly[2-(methacryloyloxy) ethyl] dimethyl-(3-sulfopropyl) ammonium hydroxide), *MEDSAH* ([2-(methacryloyloxy) ethyl] dimethyl-(3-sulfopropyl) ammonium hydroxide) monomer, *PAAc* poly(acrylic acid)

### Natural polymeric hydrogel scaffolds

#### Polysaccharides

- HA
  HA comprises linear high-molecular-weight alternating copolymers of β-(1-3)-d-glucose and β-(1-4)-2-acetamide-2-deoxy-d-glucose [35]. It is produced via *Streptococcus* fermentation or extracted from chicken comb, umbilical cord, and synovial fluid and plays an important role in the nutritional supply, lubrication, and shock absorption of joints [36, 37]. HA binds to cell surface receptors to regulate the macroscopic and microscopic environments of the tissue, thereby enabling the motility receptor and intercellular adhesion molecule-1 to perform different functions for treating arthritis pain [38]. Gan et al. [39] developed a polydopamine-modified HA double-crosslinked collagen matrix hydrogel scaffold that could induce cartilage regeneration. It also inhibited the expression of inflammatory cytokines, which activated the conversion of macrophages to the M2 phenotype and provided a favorable microenvironment for cartilage regeneration.
  HA-based injectable hydrogel systems mimic the extracellular environment and deliver anti-inflammatory drugs for cartilage regeneration [40]. A combination of peptides, cell growth factors, and stem cells with HA hydrogel scaffolds can improve the repairing ability of ACI. BMSCs combined with an HA hydrogel scaffold transplanted into an adult canine articular cartilage defect (ACD) model can significantly inhibit fibrosis and inflammation, promote cartilage regeneration at the injured site, and play a significant role in cartilage tissue repair [41]. HA also enhances the secretion of related cytokines to facilitate cartilage repair [42].
  However, the use of HA alone as a hydrogel scaffold has certain disadvantages, such as poor mechanical properties and high degradation rate, which can be overcome via chemical modification or crosslinking. For example, the carboxyl group of HA can be transformed into an amide bond using carbodiimide or carbodiimidazole, the hydroxyl group may be converted into an ether or ester, and –NHCOCH_3_ can be modified via deacetylation, amidation, and hemiacetal formation [43, 44]. Ma et al. [45] 3D printed HA methacryloyl hydrogels for treating in situ grade IV cartilage defects in rabbits as defined by the International Cartilage Repair Society and repaired the damaged cartilage sites 12 weeks later.
- Chitosan
  Chitosan, a copolymer of β-(1,4)-d-glucose and *N*-acetyl-d-glucosamine, is a natural and renewable basic polysaccharide formed by the deacetylation of chitin that exhibits good biocompatibility, adhesion, and degradability [46, 47]. Yang et al. [48] synthesized an injectable ethylene glycol–chitosan hydrogel. After in vivo experimental walking function measurements, they found that the adipose-derived stem cell (ADSC)-containing glycol chitosan/dibenzaldehyde-terminated PEG hydrogel transplanted into fresh cartilage defect areas promoted cartilage regeneration and repair. Joint injuries are typically accompanied by cartilage and subchondral bone injuries. To simulate the complex environment of different layers of natural joints, Erickson et al. [49] constructed a layered hydrogel scaffold using chitosan–HA to simulate the cartilage layer and chitosan to simulate the bone layer. After co-culturing with chondrocyte-like cells, the increased expression of genes related to cartilage formation indicated the potential applicability of this bilayer scaffold for cartilage regeneration. Xu et al. [50] used graphene oxide to modify chitosan and HA to form a hydrogel scaffold that effectively promoted the differentiation of BMSCs into chondrocytes.
  Pure chitosan hydrogels have several disadvantages, including poor mechanical properties and weak chemical stability. Crosslinking agents, alkylation agents, and fillers are typically used to improve chitosan properties [51, 52]. By modifying kartogenin with carbodiimide hydrochloride and linking it to a chondroitin sulfate scaffold and aldehyde-modified oxidized alginate compound, a system that enabled the stable release of kartogenin for chondrogenesis has been developed. Injectable hydrogels containing BMSCs have also been formulated to provide mechanical support to articular cartilage and promote cartilage differentiation [53]. By crosslinking HA and chondroitin sulfate with l-lysine diisocyanate, a hydrogel with an interpenetrating network structure was formed, enabling the stable delivery of chondroitin sulfate to promote the cartilage repair [54]. The introduced chemical crosslinking agent exhibited low toxicity with little effect on cell survival, as demonstrated by the results of toxicity tests and cell adhesion assays [53, 54].
- Alginate
  Alginate is an anionic polysaccharide long-chain copolymer comprising (1,4)-crosslinked d-mannuronic acid and (14)-crosslinked glucuronic acid. Alginate can be obtained from brown algae and *Pseudomonas* mucilage and exhibits high hydrophilicity, biocompatibility, and water solubility [55–57]. Drug-loaded alginate composite hydrogels that are applied to rat ACD models can repair cartilage tissues by preserving the chondrocyte phenotype, controlling the host inflammatory response, and effectively inhibiting ECM degradation [58]. The 3D structure of alginate hydrogels is formed via the interactions between carboxyl groups and divalent or multivalent cations. The introduction of divalent or multivalent cations enhances the biocompatibility of alginate hydrogels, rendering them highly tolerable by living tissues without causing significant immune responses or toxicity. Alginate hydrogels can be used as carriers for encapsulating chondrocytes (cartilage cells) or MSCs to promote cartilage regeneration at the defect site. Ying et al. [59] used Lentivirus–TGF-β1–EGFP-transfected BMSCs loaded with calcium alginate hydrogel to promote cartilage repair via the Smad2/3 signaling pathway. Bar et al. [60] injected the alginate saline gel encapsulated with bioactive substances into the cartilage defect site; they found that the hydrogel promoted the endogenous regeneration of cartilage by combining with TGF-β1. Alginate saline gel is widely used in tissue engineering for articular cartilage repair because of its good biocompatibility and ECM-similar structure. However, the clinical application of alginate is limited owing to its low mechanical strength, poor gel stability, and rapid drug release. Currently, the primary modification methods for improving alginate properties involve mixing natural polymers (polyvinyl alcohol, polyethylene oxide, and PCL), chemical modifiers (sodium periodate oxidizes the hydroxyl group on the glyoxal unit of sodium alginate), sulfation (replacement of alginate hydroxyl groups with sulphate salts), and graft copolymerization (alginate coupled with the carboxylic group of carbodiimide) [61, 62].

#### Poly(amino acids)

1. Collagen
  Collagen is a macromolecule comprising three intertwined polypeptide chains. It is widely present in the organic matter and connective tissue of articular cartilage, tendons, and bone, accounting for ~ 30% of the total animal protein [63]. It exhibits unique physicochemical and immunological properties, is biodegradable and biocompatible, and supports cell growth [64]. Collagen can be introduced into matrix systems, such as hydrogel scaffolds, injection media, and dispersions. Collagen scaffolds can support cell adhesion, proliferation, and migration and provide a functional niche for cartilage regeneration [11]. For example, an injectable collagen hydrogel injected into the ACD of Sprague–Dawley rats successfully induced BMSCs to differentiate into hyaline cartilage [65]. ADSCs encapsulated into a type II collagen hydrogel was transplanted into a rabbit knee joint defect model. After 12 weeks, the defect surface was neat and smooth with the surrounding native cartilage [66]. A composite scaffold comprising hydroxyapatite type I collagen and PLGA–PEG–PLGA thermal gel promoted the chondrogenesis of BMSCs [67]. In addition, the delivery of transfected MSCs with a collagen–alginate saline gel enhanced the expression of transfected SOX9, reduced the number of mast cells, and promoted MSC chondrogenesis [68]. However, pure collagen hydrogels exhibit poor mechanical properties, which limit their practical use. Therefore, chemical crosslinking can be performed using glutaraldehyde, carbodiimide crosslinking agents, or enzymes [69].
2. Silk fibroin
  Silk fibroin is a biopolymer consisting of hydrophilic sericin and hydrophobic fibroin, which possess excellent mechanical, antioxidant, and antibacterial properties as well as biodegradability; and biocompatibility [70, 71]. Fibroin hydrogels prepared via physical and chemical methods can be used to repair cartilage damage [72]. Yuan et al. [73] developed an injectable silk fibroin hydrogel via ultrasonic crosslinking and confirmed that the hydrogel promoted cartilage regeneration in a rabbit ACD model. Cui et al. [74] repaired a silk fibroin hydrogel that contained stem cells via photo-crosslinking, which promoted the long-term survival of stem cells and formation of cartilage tissues. Currently, silk–fibroin composite hydrogels are widely used in various fields. Zhou et al. [75] synthesized micro/nanocomposite hydrogels from malleated chitosan and methacrylate silk fibroin (MSF). At an MSF concentration of 0.1%, the compression modulus of the hydrogel was comparable to that of the articular cartilage. The hydrogel exhibited good biocompatibility and the ability to attach articular chondrocytes to mice. In addition, the established literature has demonstrated that the β-sheet layer of silk protein can be modified using physical and/or chemical approaches to enhance the mechanical strength of silk protein [76].
3. Gelatin
  Gelatin is a mixture of peptides produced by hydrolyzing collagen and breaking the hydrogen and covalent bonds of its triple-helical structure, which is highly water-soluble and is found in skin, tendons, ligaments and bones [77]. Gelatin is a hydrogel scaffold material widely used as a drug or cell growth factor carrier. Han et al. [78] synthesized injectable GelMA@DMA–MPC microspheres as biomimetic lubrication coatings and drug delivery carriers that could effectively protect articular cartilage and relieve inflammation. In addition, using polydopamine-coated silica nanoparticles incorporated into large-pore gelatin microsphere hydrogel scaffolds to release TGF-β3 can induce MSC differentiation into chondrocytes [79]. A gelatin hydrogel loaded with ketogenic and TGF-β1 combined with human BMSCs was injected into the focus of rat cartilage defect model to boost the formation of hyaline cartilage and subchondral bone regeneration [80]. Hydrogel-encapsulated rat ADSCs transplanted into the ACD exhibited good cartilage regeneration properties [81]. However, when used as a repair material for articular cartilage, gelatin has several disadvantages, such as low thermal stability and mechanical strength [82]. They can be mitigated by modifying the reactive functional groups of gelatin side chains, such as -NH_2_, -OH, and -COOH. For example, a polyamide amine was added to a methacrylate (GelMA) hydrogel to prepare ADSC hydrogel scaffolds with uniform structures, high stability, and good mechanical properties, as shown in Table 1 [83].

### Synthetic polymeric hydrogel scaffolds

#### PEG

PEG is a water-soluble linear polyether that is polymerized from ethylene oxide and exhibits good biocompatibility and excellent mechanical properties. It is widely used as a scaffold material in tissue engineering [84]. Platelet-rich plasma (PRP) has attracted considerable interest in the treatment of ACI owing to the presence of numerous growth factors and proteins [85]. However, its practical applicability and efficacy are limited by the action of lyases and short-term release of bioactive factors. Censi et al. [86] prepared a PEG-based inter-permeable network hydrogel that improved the in vitro stability of PRP and significantly increased its degradation time and storage modulus. The hydrogel could regulate the release of platelet-derived growth factors (PDGFs) and TGF-β1 to improve tissue adhesion. The encapsulation of lyophilized PDGF in a PEG hydrogel induces chondrocyte proliferation and inhibits anti-inflammatory gene expression [87]. In addition, because the chemical composition of PEG (–(CH_2_CH_2_O)_n_–) does not contain a biological recognition site, PEG should be modified to improve the inertia and cellular stimulus response of the hydrogel biomimetic network structure. A biomimetic hydrogel scaffold containing cartilage-protective factors can promote the homing and differentiation of endogenous MSCs into chondrocytes to repair defective cartilage tissues [88]. siRNA–polyethylene glycol diacrylate (PEGDA) hydrogels were fabricated using a photo-crosslinked bio-ink of silk methacrylate and PEGDA mixed with chondrocytes via 3D bioprinting. They demonstrated reliable rheological properties, printability, and excellent mechanical and degradation characteristics. However, because of the poor biocompatibility of PEGDA, its low concentrations can be mixed with GelMA or alginate saline gel with chondrocytes for 3D printing to produce cartilage hydrogel scaffolds with high mechanical strength and good biocompatibility [89].

#### PLA

PLA is an aliphatic polymer formed via lactic acid condensation. It has two optical forms: poly-l-lactic acid and poly-d-lactic acid. PLA is primarily a synthetic polymer, and only PLA containing poly-l-lactic acid includes natural biopolymers [90]. As a biodegradable material, it is widely used for repairing damaged tissues, including articular cartilage. PLA is mainly used for cartilage repair through either injection or microsphere-based delivery. Bone morphogenetic protein (BMP) is an important component of the TGF-β family, which is a major factor promoting cartilage formation. Focal condylar cartilage defects in rabbits were treated with PLGA-encapsulated BMP-2 and BMSC injectable hydrogel scaffolds, which stably delivered cells and growth factors and repaired 85% of the injury sites after transplantation [91]. Jin et al. [92] used PLGA microspheres to deliver BMP-7 and synovium-resident mesenchymal stem cells (synMSCs) into a rabbit cartilage defect mold to increase the contents of type II collagen and proteoglycans and induce cartilage regeneration. In addition, loaded Kartogenin was grafted to the injury site via injection using a cartilage defect model for a minipig. A 12-month follow-up study confirmed that the hydrogel exerted a strong repair effect for the treatment of full-length cartilage defects [93]. The intra-articular release of a leptin inhibitor using PDLLA-based thermosensitive injectable hydrogel activates mTORC1 and induces chondrocyte autophagy for articular cartilage regeneration [94]. Although PLA has a wide range of practical applications, it has certain limitations, such as low strength, brittleness, slow degradation, and poor hydrophobic impact toughness. The addition of modified lignin to PLA significantly improved the impact strength and thermal stability of the material [95]. Furthermore, Zhang et al. [96] prepared an amphiphilic polymer (PLGA–g-PCL)-based hydrogel scaffold with high strength and elasticity by polymerizing the short-chain hydrophobic region of PCL with the hydrophilic end of PLGA, and the hydrogel-loaded adipose-derived stem cells demonstrated good meniscal regeneration properties in an animal model of meniscal defects.

#### PVA

PVA is a synthetic polymer with good biocompatibility, excellent mechanical properties, and low friction, which is obtained by the alcoholysis of polyvinyl acetate [97–99]. It is a promising material for cartilage tissue engineering and synthetic polymer approved by the U.S. Food and Drug Administration. The low friction of PVA enables its potential use for simulating articular cartilage. By measuring the coefficient of friction between a PVA hydrogel and natural articular cartilage, it was found that load and velocity were the key factors affecting its magnitude, leading to superficial adhesive wear [100]. Yang et al. [101] developed a (PVA)–poly(2-acrylamido-2-methyl-1-propanesulfonic acid sodium salt) (PAMPS) (BC–PVA–PAMPS) hydrogel, which exhibited the same tensile strength as that of the collagen in cartilage, while its penetrating resilience was identical to that of aggregated proteoglycans. Moreover, the coefficient of friction of the synthesized hydrogel was 40% lower than that of natural articular cartilage, and its abrasion resistance was 4.4 times greater than that of pure PVA hydrogel. PVA–amphiphilic polymer (PVA–PMEDAH) hydrogel scaffolds also exhibited good mechanical and frictional properties [102], while pure PVA hydrogels possessed weak mechanical properties. A novel PVA/CS porous hydrogel demonstrated excellent mechanical characteristics and was non-cytotoxic. It was transplanted into a rabbit osteochondral defect model showing a high healing efficiency [103]. Bichara et al. [104] developed PVA–poly(acrylic acid) (PAAc) hydrogel grafts in a New Zealand white rabbit OC defect model; the prepared hydrogel retained its high water content with intact meniscus tissue surfaces after 12 weeks versus 24 weeks of grafting.

Crosslinking agents are extensively utilized in both natural and synthetic polymer-based scaffolds. They represent chemical compounds containing two or more reactive ends that promote bonding between macromolecules, leading to the formation of a 3D mesh structure. With the development of hydrogels, crosslinkers have been extensively used in the field of hydrogel biomedical applications, because they can significantly improve the biomechanical properties of tissues [105]. The main crosslinking agents currently utilized in hydrogels include carbodiimide, glutaraldehyde, and epoxy compounds. The HADN hydrogel obtained by coupling HA and dopamine with carbodiimide exhibited good cartilage lubrication properties and effectively protected cartilage tissues [106]. HADN coupled with a crosslinker and glutathione demonstrated good biocompatibility with chondrocytes [107]. When human adipose-derived stem cells (hADSCs) were cultured in pericardial extracts with 0.1% glutaraldehyde crosslinked to collagen, the cell growth rate remained above 86% of the normal control group, demonstrating basic appropriate biocompatibility [108].

However, the crosslinking agent is a chemical reagent, and its toxicity should not be overlooked as it may cause tissue calcification, the development of inflammation in vivo, and mechanical damage during handling. The addition of crosslinking agents may also affect the purity of the prepared samples because of their residual amounts. The hydrogel precursor material was obtained by oxidizing alginate using sodium periodate, and residual sodium periodate was removed from the precursor via dialysis at the end of the reaction [109, 110]. Physical crosslinking can also be used when the requirements for material properties are not as stringent.

In summary, the natural and synthetic polymer-based scaffold materials have been successfully used for the repair of ACI. As scaffold materials for tissue engineering, both the natural and synthetic materials exhibited their own strengths and weaknesses (Tables 1, 2, and 3). Synthetic polymers have few inherent biological properties of natural polymers. However, their mechanical strength, degradation, and biological responses can be easily controlled through structural modification. Furthermore, synthetic polymers-based scaffolds exhibit more outstanding properties (including processability, batch–batch consistency, and flexibility) as compared with those of natural polymers-based scaffolds.

## Injectable hydrogel scaffolds for articular cartilage repair

Injectable hydrogels have become a hot research topic. Injectable hydrogel can transport cells, growth factors, peptides and other substances to the damaged cartilage site, such as the current treatment of OA through minimally invasive injection of HA hydrogel into the joint cavity, which reduces the trauma caused by the surgery and the occurrence of related complications. Currently used for the preparation of injectable hydrogels include natural and synthetic polymers. The main cross-linking modes for injectable hydrogels are physical cross-linking such as ionic bonding, hydrogen bonding, hydrophobic interactions and van der Waals and chemical cross-linking such as Schiff base reaction, photopolymerization, Micheal addition, enzyme-mediated reaction, and click chemistry.

Physical cross-linking usually has better biocompatibility, because it does not involve the use of chemical cross-linking agents, can be degraded under certain conditions, has good environmental adaptability, and its mechanical strength is usually lower than that of chemically cross-linking hydrogels, but it can be improved by optimizing the preparation and structural design under certain conditions. Yu et al. [111] constructed a drug-carrying, injectable hydrogel that not only maintains the phenotype of chondrocytes, but also modulates the host's inflammatory response by means of thermal response. An injectable and biodegradable piezoelectric hydrogel made of short electrospun poly-l-lactic acid nanofibers within a collagen matrix, which generates local electrical signals on its own under ultrasound activation to drive cartilage healing [112]. In addition, the in vivo cartilage regeneration ability of US–SF hydrogel was confirmed after subcutaneous administration in nude mice and in situ injection in a rabbit osteochondral defect model using SF injectable hydrogel prepared by a one-step ultrasonic cross-linking method [73].

Unlike physical cross-linking, chemical cross-linking uses chemical cross-linking agents, which involves the use of compounds that form chemical cross-links through chemical reactions such as addition and condensation to obtain a strong cross-linked network. Commonly used chemical cross-linking agents include glutaraldehyde, formaldehyde, and genipin. This method has the advantage of simplicity of operation, but may leave unreacted monomers or cross-linking agents that can affect biocompatibility. For example, crosslinked mercapto-functionalized hyaluronic acid and hyperbranched poly(ethylene glycol) polyacrylate macromolecules prepared by the mercapto-en-Michael addition reaction are injectable hydrogels. The cartilage-derived progenitor cells (CPC) embedded in them maintain high levels of cell viability, proliferative properties, and anti-inflammatory capacity [113]. Aisenbrey et al. encapsulated MSCs in a photo-clickable polyethylene glycol hydrogel containing chondroitin sulfate and RGD. hMSCs in this hydrogel inhibited the terminal differentiation of chondrogenic differentiation. chondroitin sulfate produces supportive MSC chondrogenic physicochemicals up-regulate the p38 MAPK pathway and down-regulate the SMAD 1/5/8 signaling pathway to reduce mast cell formation [114].

With continuous innovation in research methods, the use of a combined physical and chemical approach (dual cross-linking method) shows great promise in the field of improving injectable hydrogels for cartilage damage repair. Hydrogels prepared by the dual crosslinking method have higher mechanical strength and better stability compared to the single crosslinking method. For the treatment of articular cartilage defects in hemophilia, Bin Chen's group developed an adhesive hydrogel with dynamic Schiff alkali and hydrogen bonding, which exhibits excellent injectability, wet tissue adhesion and hemostatic properties. Inspired by articular cartilage matrix [115]. O’Shea et al. [116] developed a composite hydrogel of HA and collagen for injection and 3D printing (3DP) with improved physicochemical properties for delivery of autologous cells and matrix components to the injured joint site via minimally invasive arthroscopy. Behan et al. [117] developed an injectable hydrogel composed of decellularized extracellular matrix (dECM) methacrylate and gelatin with excellent performance in repairing damaged joints.

## Progress and challenges in current clinical applications of hydrogels

Hydrogels have been extensively used in experimental studies for the treatment of ACI; however, several studies remain in the pre-clinical stage and have not been translated into clinical applications yet. Nevertheless, the hydrogels currently used in clinical practice have demonstrated excellent repair properties. For example, Park et al. [118] encapsulated allogeneic cord blood-derived MSCs in HA hydrogels and transplanted them into patients with knee OA. No apparent overgrowth, ossification, or tumor formation occurred during the 7-year follow-up period. Wolf et al. [119] treated patients with focal ACDs in the knee joint using ChonDux hydrogel scaffolds (A hydrogel scaffold formed by bonding chondroitin sulfate to PEG hydrogel) and evaluated defect recovery via magnetic resonance imaging conducted 3–24 months after the surgery. The obtained results revealed that 94.2% ± 16.3% of cartilage defects were recovered within 24 months and that the hydrogel maintained a continuous repair effect on cartilage tissue. In addition, Niemeyer et al. exerted a strong repair effect of the hydrogel-based autologous chondrocytes for the treatment of focal full-thickness cartilage defects in the knee joint over 2 years [120]. In another study, an autologous chondrocyte–hydrogel mixture was injected into the hip joint. After 12 months, the joint activity level of the patient increased, quality of life improved, and pain symptoms decreased [121]. Blanka Sharma’s team [122] used chondroitin sulfate adhesive in combination with PEGDA hydrogel applied to 15 patients with focal cartilage defects of the medial femoral condyle. Magnetic resonance imaging showed that the treated patients achieved significantly higher levels of tissue filling compared to the control group. Magnetic resonance spin–spin relaxation time (T2) showed an increase in water content over time and an increase in tissue organization. Patients who received treatment had reduced pain compared to controls, while knee function scores increased to similar levels between the two groups over the 6 months assessed. Recently, De Faro Silva et al. [123] developed a natural hydrogel nano-encapsulated drug containing Carbopol^®^ for the treatment of knee OA in female, and the subsequent follow-up found significant improvements in the quality of life and joint function of patients. A study of type I collagen hydrogel autologous chondrocyte implantation (CaReS) for repair of cartilage defects in the knee placed 116 patients who underwent CaReS implantation at 9 different centers in the knee and were evaluated for International Knee Documentation Committee (IKDC) scores, pain scores (Visual Analog Scale [VAS]), SF-36 score, overall treatment satisfaction, and IKDC functional status. At postoperative follow-up over a period of six consecutive years, the overall pain level decreased significantly from 6.7 ± 2.2 preoperatively to 3.2 ± 3.1 at the final follow-up visit. There was also a significant increase in both components of the SF-36 score [124]. These studies provide preliminary evidence of the potential value of hydrogels for the treatment of cartilage injuries (Table 4).

**Table 4:** Clinical study of hydrogels strategies in the repair of articular cartilage injury

Intervening measure	Research type	Studies/patients (*n*)	Treatment outcomes	Disease type	References
Complexes of allogeneic human umbilical cord blood-derived mesenchymal stem cells (hUCB–MSCs) and hyaluronic acid hydrogels [Cartistem]	Case series	7 patients	Improved clinical outcomes remained stable at 7-year follow-up, with no cases of osteogenesis or tumorigenesis	Osteoarthritic	[118]
ChonDux Hydrogel(A hydrogel scaffold formed by bonding chondroitin sulfate to PEG hydrogel)	Case series	18 patients	Promotes stable repair of full-layer articular cartilage defects within 24 months	Focal articular cartilage defect of the knee joint	[119]
Albumin–hyaluronic acid hydrogel	Prospective, multicenter, single-arm, phase III clinical trial	100 patients	Two years after ACI treatment, 93% of patients were KOOS responders having improved by ≥ 10 points compared with their pre-operative level	Large cartilage defect of the knee joint	[120]
Injectable autologous chondrocyte transplantation products	Prospective therapeutic study	13 patients	Increased activity levels, improved quality of life and reduced pain after 12 months of follow-up	Hip cartilage defects	[121]
Poly(ethylene glycol) diacrylate (PEGDA) hydrogel	Pilot clinical trial	18 patients	Reduced pain in patients receiving treatment	Focal cartilage defect of the medial femoral condyle	[122]
Pharmaceutical formulations containing Carbopol® hydrogels	double-blind randomized controlled trial	18 patients	Increased muscle strength in knee extensors and flexors at 21 days in women treated with PeONC	Osteoarthritis	[123]
New Type I Collagen Hydrogel (CaReS)	Prospective multicenter study	118 patients	Five-year follow-up results show that the technique significantly improves pain levels	Articular cartilage defects in the knee	[124]

Currently, treatment of ACI using commercialized hydrogels remains challenging. Our research on hydrogels is not deep enough, and we are still in the primary stage of understanding the effects of hydrogel composition, morphology, structure and physicochemical properties on cartilage repair. Nowadays, most of the clinical trials of hydrogels are conducted in small batches, and the progress for large-scale use is slow. In addition, besides the properties of the material itself, the production process of hydrogel is also crucial. The properties of hydrogel materials are affected by many factors. For example, temperature, pH and ionic strength of the hydrogel material also affect its mechanical properties. Nowadays, the mechanical properties and stability of hydrogels can be improved to a certain extent by introducing nanomaterials or performing cross-linking reactions, but most of the hydrogels developed so far still have the disadvantages of low strength and brittleness. In addition, cost control needs to be considered to realize the commercial application of hydrogels. It is necessary to reduce the production cost by optimizing the production process and improving the production efficiency to make the products competitive in the market. In this regard, whether hydrogel can enter the market smoothly depends on the maturity of its technology in the first place. Researchers are needed to ensure that the performance of hydrogel is stable and reliable and meets the needs of different application fields through a large number of experiments and data verification.

## Conclusion and future perspectives

ACI are typically caused by coagulation, inflammation, blood infiltration, aging, obesity, trauma, and other factors that lead to its biochemical decomposition and physical deterioration. ACI can result in ligaments, menisci, soft tissue injuries, and other injuries that produce a serious impact on the spirit and lives of patients. Previous studies have shown that hydrogels exerted strong repair effects for the treatment of ACI. In this review, the sources, applications, modifications, advantages, and disadvantages of hydrogels are discussed. Compared with the traditional methods for repairing damaged cartilage, hydrogels have several advantages. In addition to their excellent properties, such as biocompatibility and degradability, different types of hydrogels can be designed for various applications, such as injectable hydrogels for easy cell transplantation to defect sites. Recently, a bilayer hydrogel scaffold has been designed based on bone and cartilage stratification characteristics. To enable the in situ regeneration of cartilage, drugs, growth factors, or stem cells can be used to induce the formation of cartilage on the hydrogel scaffold [35, 60, 61, 76, 77].

Although hydrogels are advantageous for cartilage repair, they also have several disadvantages owing to their inherent characteristics. Natural polymer hydrogel scaffolds exhibit low mechanical strengths and high degradation rates, which are not conducive to the differentiation of MSCs into chondrocytes and formation of new cartilage tissues. For example, the mechanical properties of HA hydrogel scaffolds are poor, and their degradation rates in vivo are high, while gelatin has low thermal stability. Synthetic hydrogels possess high mechanical strengths; however, their cell adhesion properties and biological safety remain insufficient. The PEG hydrogel biomimetic network structure is inert and does not respond well to cellular stimuli. PLA exhibits a low degradation rate, hydrophobicity, and impact toughness.

Based on the distinct characteristics of these materials, the prepared hydrogels have certain advantages and disadvantages. Researchers can combine the advantages of different materials based on their excellent properties to fabricate new hydrogels with stable performance, excellent mechanical properties and biocompatibility, strong cartilage adhesion, and good biological activity. Owing to these advantages, hydrogels have good application prospects for the treatment of cartilage injuries.

In summary, although hydrogels have considerably advanced the field of ACI treatment, several issues limit their practical utilization. Researchers should continue to investigate various topics, such as the therapeutic effect of hydrogels on cartilage injury, optimal hydrogel transplantation time, escape of stem cells encapsulated in hydrogels during binding, methods for controlling growth factors, and drug release rates. With the advancement of the related research studies, hydrogels can be potentially used as medications and cell carriers for effectively healing cartilage abnormalities.

## Data Availability

No datasets were generated or analysed during the current study.
